# Incrimination of *Phlebotomus kandelakii* and *Phlebotomus balcanicus* as Vectors of *Leishmania infantum* in Tbilisi, Georgia

**DOI:** 10.1371/journal.pntd.0001609

**Published:** 2012-04-03

**Authors:** Ekaterina Giorgobiani, Phillip G. Lawyer, Giorgi Babuadze, Nato Dolidze, Ryan C. Jochim, Lamzira Tskhvaradze, Konstantin Kikaleishvili, Shaden Kamhawi

**Affiliations:** 1 National Center for Disease Control and Public Health, Tbilisi, Georgia; 2 Laboratory of Parasitic Diseases, National Institute of Allergy and Infectious Diseases, National Institutes of Health, Bethesda, Maryland, United States of America; 3 Laboratory of Malaria and Vector Research, National Institute of Allergy and Infectious Diseases, National Institutes of Health, Rockville, Maryland, United States of America; Hebrew University-Hadassah Medical School, Israel

## Abstract

A survey of potential vector sand flies was conducted in the neighboring suburban communities of Vake and Mtatsminda districts in an active focus of visceral Leishmaniasis (VL) in Tbilisi, Georgia. Using light and sticky-paper traps, 1,266 male and 1,179 female sand flies were collected during 2006–2008. Five *Phlebotomus* species of three subgenera were collected: *Phlebotomus balcanicus* Theodor and *Phlebotomus halepensis* Theodor of the subgenus *Adlerius*; *Phlebotomus kandelakii* Shchurenkova and *Phlebotomus wenyoni* Adler and Theodor of the subgenus *Larroussius*; *Phlebotomus sergenti* Perfil'ev of the subgenus *Paraphlebotomus*. *Phlebotomus sergenti* (35.1%) predominated in Vake, followed by *P. kandelakii* (33.5%), *P. balcanicus* (18.9%), *P. halepensis* (12.2%), and *P. wenyoni* (0.3%). In Mtatsminda, *P. kandelakii* (76.8%) comprised over three fourths of collected sand flies, followed by *P. sergenti* (12.6%), *P. balcanicus* (5.8%), *P. halepensi*s (3.7%), and *P. wenyoni* (1.1%). The sand fly season in Georgia is exceptionally short beginning in early June, peaking in July and August, then declining to zero in early September. Of 659 female sand flies examined for *Leishmania*, 12 (1.8%) specimens without traces of blood were infected including 10 of 535 *P. kandelakii* (1.9%) and two of 40 *P. balcanicus* (5.0%). Six isolates were successfully cultured and characterized as *Leishmania* by PCR. Three isolates from *P. kandelakii* (2) and *P. balcanicus* (1) were further identified as *L. infantum* using sequence alignment of the 70 kDa heat-shock protein gene. Importantly, the sand fly isolates showed a high percent identity (99.8%–99.9%) to human and dog isolates from the same focus, incriminating the two sand fly species as vectors. Blood meal analysis showed that *P. kandelakii* preferentially feeds on dogs (76%) but also feeds on humans. The abundance, infection rate and feeding behavior of *P. kandelakii* and the infection rate in *P. balcanicus* establish these species as vectors in the Tbilisi VL focus.

## Author Summary

Visceral *Leishmania*sis (VL) is a public health problem in Tbilisi, capital of the Republic of Georgia. VL is caused by *Leishmania* parasites and dogs represent the main infection reservoirs. VL is transmitted among humans and dogs by sand fly bites. Here, we carried out a three-year survey to assess the sand fly species in two communities within the VL focus of Tbilisi in the districts of Vake and Mtatsminda. We collected five sand fly species, and the most abundant was *Phlebotomus kandelakii*. We found live parasites in the midgut of *P. kandelakii* and another species, *P. balcanicus*. Using molecular techniques we identified the parasites as *Leishmania infantum*. We also found that these sand fly isolates shared a high identity to parasites isolated from dogs and humans in the same focus. This incriminates *P. kandelakii* and *P. balcanicus* as vectors of VL in this focus. The source of the blood meals in fed flies revealed that *P. kandelakii* preferentially feeds on dogs but also feeds on humans. The abundance, infection rate and feeding behavior of *P. kandelakii* and the consistently higher infection rate of *P. balcanicus* compared to *P. kandelakii* incriminate these species as primary vectors in the Tbilisi VL focus.

## Introduction

Historically, visceral leishmaniasis (VL) in Georgia has been characterized by sporadic cases chiefly in eastern mountainous districts indicative of an endemic situation [1], [2]. It wasn't until the 1990s that VL due to *Leishmania infantum* was recognized as a significant public health problem in the Republic of Georgia [3]. From 1990–2007 there was a resurgence of the disease, with 1,414 cases reported involving an 18-fold increase from 10–12 cases per year in the 1990's to 182 cases in 2007 [Official statistical records, National Centers for Disease Control and Public Health (NCDCPH), Tbilisi, Georgia]. Sixty percent of these cases occurred within the capital city of Tbilisi, a modern city of 1.2 million inhabitants. In response to this alarming increase, a three-phase program was initiated to gain a better understanding of the epidemiologic cycle of the disease in this focus, including active surveillance in children, dogs and potential vector sand flies. Surveillance of children showed that 7.3% of 4,250 children aged 1–14 years were seropositive for *Leishmania* at the baseline survey, and 6.0% became seropositive over one year [4]. Risk of infection was associated with living in areas where clustered flying insects and stray dogs were observed. For dogs, the major reservoir of *L. infantum* infection, 18.2% of 588 domestic and 15.3% of 718 stray dogs surveyed were seropositive [4].

Among about 20 sand fly species that play a significant role in transmission of *Leishmania* parasites in the Mediterranean basin, members of the subgenus *Larroussius* represent the most important vectors of *L. infantum* [5], [6]. In countries of the former Soviet Union, members of the subgenera *Larroussius* and *Adlerius*, such as *Phlebotomus brevis* Theodor & Mesghali and *Phlebotomus perfiliewi transcaucasicus* Perfil'ev in Azerbaijan, *Phlebotomus longiductus* Parrot in Uzbekistan and Kazakhstan, and *Phlebotomus kandelakii* Shchurenkova in Georgia, were suspected as vectors of *L. infantum* but none were incriminated [5], [7]. Recent investigations on vectors of VL in northwestern Iran (Ardebil and Fars provinces) and East Azerbaijan found *P. perfiliewi transcaucasicus*, *P. kandelakii*, and *Phlebotomus* (*Adlerius*) sp., naturally infected with parasites belonging to the *Leishmania donovani* complex by PCR [8]–[10].

Studies on the distribution, seasonality and behavior of sand flies in disease foci in Georgia have not been carried out for the past 20 years. Lemer [1] conducted an investigation of potential vector sand flies in eastern Georgia from 1942–1952. He reported on the specific composition of sand fly populations in four localities and on the seasonal occurrence and epidemiological importance of *Phlebotomus kandelakii* Shchurenkova and *P. chinensis balcanicus* Newstead (*Phlebtomus balcanicus*, Theodor), which were the predominant species and the only ones common to all four localities. In one of the localities, it was observed that both species were infected with leptomonads (promastigotes), the infection rate being 3.7 percent [1]. Although such evidence casts suspicion on these species as vectors of *L. infantum* it does not prove the role of a vector [5]. A more recent review listed fourteen species of *Phlebotomus* sand flies identified in previous entomological surveys, all from eastern Georgia: *Phlebotomus papatasi* Scopoli of the subgenus *Phlebotomus; Phlebotomus caucasicus* Marzinowsky, *Phlebotomus mongolensis* Sinton, *Phlebotomus sergenti* Perfil'ev and *Phlebotomus jacusieli* Theodor of the subgenus *Paraphlebotomus; P. kandelakii, Phlebotomus tobbi* Adler & Theodor, *Phlebotomus syriacus* Adler & Theodor, *P. transcaucasicus* and *Phlebotomus wenyoni* Adler & Theodor of the subgenus *Larroussius*; and *Phlebotomus simici* Nitzulescu, *Phlebotomus halepensis* Theodor, *Phlebotomus chinensis* Newstead, and *Phlebotomus balcanicus* Theodor of the subgenus *Adlerius* [11]. *P. kandelakii*, *P. balcanicus* and *P. sergenti* were the predominant species, with *P. kandelakii* being the most abundant [11]. The involvement of these sand flies in the transmission of VL was not addressed in any of these surveys.

Here, we report on the species diversity, relative abundance and spatial and temporal distribution of phlebotomine sand flies within an active VL focus in Tibilisi, Georgia. We also provide compelling evidence incriminating *P. kandelakii* and *P. balcanicus* as vectors of *L. infantum* in this focus, report their natural *Leishmania* infection rates and demonstrate that isolates obtained from these wild-caught specimens are identical to *L. infantum* isolates obtained from humans and canines in the same focus. Additionally, this is the first study in which live parasites were isolated from the sand fly species *P. kandelakii* and *P. balcanicus* and characterized as *L. infantum*.

## Materials and Methods

### Ethics Statement

Oral consent was obtained from heads of compounds chosen for collection of sand flies in Vake and Mtatsminda. Light traps and sticky traps were only placed outside houses, in courtyards, animal pens and shelters. Flies fed on human volunteers were obtained from activities related to a project addressing human immune responses to sand fly saliva. This project was approved by the Walter Reed Army Medical Center Human Use committee (protocol # 355023). All the subjects provided written informed consent. Acquisition of dog blood was done under animal protocol LMVR 7E approved by the NIAID DIR ACUC committee that adheres to the U. S. Government Principles for the Utilization and Care of Vertebrate Animals Used in Testing, Research, and Training and maintains animals in accordance with the PHS Policy on Humane Care and Use of Laboratory Animals, the Guide for the Care and Use of Laboratory Animals, and the Animal Welfare Act and Animal Welfare Regulations user guidelines.

### Description of Study Sites

**Figure 1:**
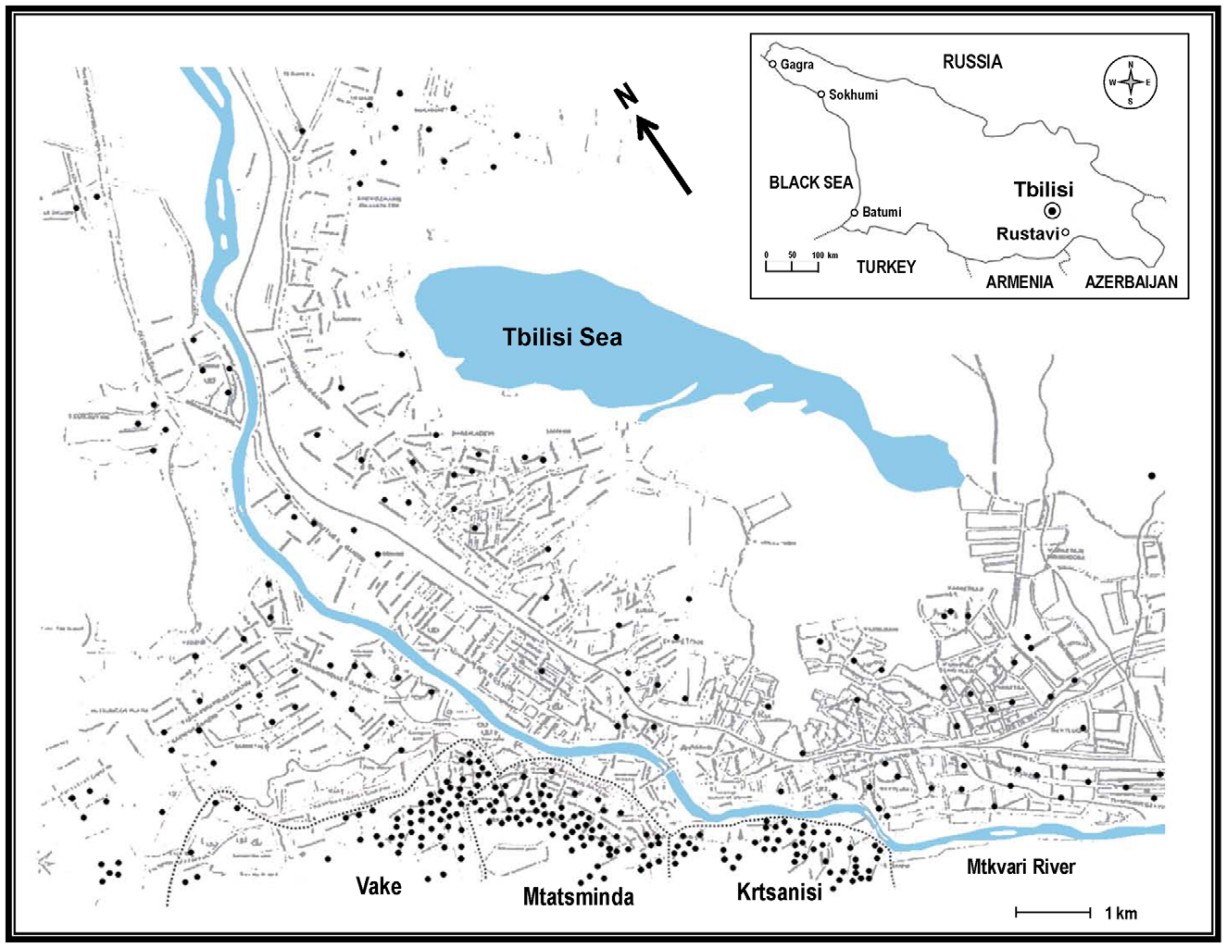
Map of the visceral leishmaniasis (VL) focus in Tbilisi, Georgia. The districts of Vake, Mtatsminda and Krtsanisi are indicated. • = human VL case sites. Inset shows the geographical location of Georgia relative to neighboring countries and the position of its capital Tbilisi.

The city of Tbilisi is situated in a narrow valley on the banks of the Mt'k'vari (Kura) River, flanked on the east and west by steep hills. The neighboring communities of Vake and Mtatsminda, from whence cases of VL are reported, are located on the western flank of the valley overlooking the city (Fig. 1). Homes in these communities are modest, mostly of brick or stone construction. Most are within fenced or walled compounds containing courtyards, trees and orchards, gardens, grape arbors and pens for animals including dogs, chickens and rabbits, thus offering a diversity of blood meal sources as well as protected microhabitats suitable as resting and breeding sites for sand flies. Windows are unscreened, permitting sand flies free access to residents.

### Vector Surveillance

#### Routine sand fly collection

A longitudinal survey was conducted during the summers of 2006–2008 to assess the diversity and relative abundance of potential *Leishmania* vectors in the Tbilisi focus and to examine their seasonal population profiles. Sand flies were collected on a monthly basis from early June through mid September during each of the survey years. Seven family compounds, all previous or active case sites, were selected for surveillance in each of the two study communities, Vake and Mtatsminda. Fourteen CDC miniature light traps (John W. Hock Company, Gainesville, FL 32606, USA) were used to collect sand flies during three consecutive nights per month in each community. Traps were placed outside houses only, two per family compound, in covered or protected habitats such as in or near animal pens, in courtyards adjacent to houses or under the eaves of buildings. Traps were suspended so that the attracting light source was approximately 1.5 meters above the ground and were operated from one hour before sunset until one hour after sunrise the following morning. Three to five sticky-paper traps (A4 bond paper coated with castor oil) were placed in cracks and crevices and other likely sand fly resting sites within 10 meters of each light-trap. The trap locations were fixed throughout the study period. The light traps and sticky traps were retrieved each morning and taken to the NCDCPH laboratory for processing.

#### Specimen processing and identification

Sand flies captured in light traps were removed from the collecting bags with a mouth aspirator and transferred to a dish of soapy water to immobilize them and for surface cleansing. Flies captured on sticky paper traps were removed with a fine-haired paint brush and transferred to soapy water to remove the castor oil. The sand flies were then transferred, one collection at a time, to clean distilled water to await identification and/or dissection and examination for *Leishmania* parasites. Each trap's collection was kept separate from all other collections and clearly labeled to ensure that individual flies could be traced to their specific collection site.

To prepare sand flies from routine seasonal surveillance for identification, male and female specimens were removed from the distilled rinse water and daubed lightly on a piece of tissue paper to remove any water droplets and then transferred to a 10% lacto-phenol clearing solution (Bioquip®, Rancho Dominguez, CA, USA) for at least an hour, but usually overnight, to render critical morphological features in the head and tip of the abdomen more visible by microscopic examination. Flies were dissected according to the methods of Young and Duncan [12] and identified to species using the dichotomous keys of Artemiev [7], [13]. Following identification, permanent mounts of selected male and female specimens of all collected species were made in polyvinylalcohol resin (PVA, Bioquip®, Rancho Dominguez, CA, USA) and are available as a reference collection.

### *Leishmania* Surveillance

#### Examination of flies for *Leishmania*

In late July and early August of 2008 and again in late July of 2010, during the period of highest population densities, sand fly collections were made separate from routine seasonal surveillance for the sole purpose of dissecting and examining females for natural *Leishmania* infections. Female sand flies were removed from traps and transferred to soapy water solution to immobilize them and for surface cleansing; then they were rinsed in clean distilled water and soaked for about 10 minutes in 1% sodium hypochlorite solution to disinfect them. Flies were dissected using sterile dissecting needles on a sterile microscope slide in a drop of sterile phosphate-buffered saline (PBS) according to the method of Lawyer et al. [14]. The terminal two segments of the abdomen containing the spermathecae were separated from the gut and transferred to a fresh drop of sterile PBS on a clean slide for identification. A sterile, glass cover slip was placed over each gut to facilitate examination under a compound microscope. The blood feeding status of each female was recorded.

#### Isolation of *Leishmania* parasites

When a fly was found infected, the cover slip was carefully removed with forceps and the entire gut including the diverticulum (crop) was transferred with a sterile dissecting needle into a fresh drop of sterile PBS. The isolate was then aspirated into a sterile, 1-cc tuberculin syringe containing 100 µl sterile complete M199 tissue-culture medium (cM199) composed of 20% heat-inactivated fetal bovine serum, 100 U/ml penicillin, 100 µg/ml streptomycin, 2 mM L-glutamine, 40 mM HEPES, 0.1 mM adenine (in 50 mM HEPES), 5 mg/ml hemin (in 50% triethanolamine) and 1 mg/ml 6-biotin and injected through the alcohol-sterilized vinyl top of an injection vial containing 1 ml of sterile blood agar solid medium (3% Bacto-agar and 10% defibrinated rabbit blood) overlaid with 1 ml sterile cM199. The vial was labeled with the fly number and identification, trap number and collection date so that the isolate could be traced to its vector and collection site. Vials containing isolates were held at room temperature for 24 hours to initiate growth and subsequently stored at 4°C to retard growth until the samples could be taken to the National Institutes of Health in the United States for expansion and identification. Primary parasite isolates were removed from the injection vials in a laminar-flow hood and expanded into 5 ml of cM199 for speciation.

#### Characterization of *Leishmania* isolates

Genomic DNA was extracted from successfully cultured sand fly isolates using the DNeasy tissue kit (Qiagen Inc., Valencia, CA) following the manufacturer's instructions. PCR analysis with primers Uni21 (5′ GGG GTT GGT GTA AAA TAG GCC 3′) and Lmj4 (5′ CTA GTT TCC CGC TCC GAG 3′) were used to confirm the identity of the isolates as *Leishmania* parasites [15]. These primers amplify a *Taq*I fragment of a kinetoplast minicircle DNA [16]. The 25 µl reaction mixtures contained 12.5 µL of FastStart PCR Master (Roche), 1 µL of each primer (10 µM), and 5 µL of parasite DNA (∼20 ng). Conditions for the amplification were as follows: initial denaturation at 94°C for 5 minutes and 35 cycles of 94°C for 1 minute, 60°C for 1 minute and 72°C for 1.5 minutes. A final elongation at 72°C for 10 min was followed by storage at 4°C. The PCR products were separated in 1.4% agarose gel alongside a TrackIt 100 bp DNA ladder (Invitrogen, Carlsbad, CA), stained with cyanine dye and visualized by ultraviolet light. *L. infantum* (MHOM/ES/00/UCM-1) and *L. major* MHOM/IL/80/Friedlin were used as reference strains.

Further speciation of the *Leishmania* parasites as *L. infantum* from representative isolates from *P. kandelakii* and *P. balcanicus* was carried out by amplification of the 70 kDa heat-shock protein gene (*HSP70*). Amplification of *HSP70* was performed with primers HSP70sen (5′-GACGGTGCCTGCCTACTTCAA-3′) and HSP70ant (5′-CCGCCCATGCTCTGGTACATC-3′) [17]. The PCR was completed in a volume of 50 µl with the AccuPrime™ *Taq* DNA Polymerase System (Invitrogen) and contained the following: 5 µl of 10× PCR Buffer II, 1 µl of each primer (10 µM each), 50 ng of template DNA, 1 µl of *Taq* DNA polymerase and 16 µl of H_2_O. The samples were incubated at 94°C for 2 minutes followed by 35 cycles under the following conditions: 94°C for 30 seconds, 60°C for 30 seconds, 68°C for 1.5 minutes. The amplified 1422 bp products were separated by electrophoresis through 1.2% agarose and visualized using SYBR Safe DNA gel stain (Invitrogen). The excised bands were extracted with Ultrafree-DA DNA Extraction (Millipore) and cleaned with three washes of ultrapure H_2_O through a YM-30 Microcon filter (Millipore). The cleaned products were ligated into pCR 4-TOPO (Invitrogen) and used to transform One Shot TOP10 competent *Escherichia coli*. Colonies were screened by PCR with HSP70sen and HSP70ant primers. Positive colonies were grown overnight and the plasmids purified with the PureLink Quick Plasmid Miniprep Kit (Invitrogen). The plasmids were sequenced bidirectionally with M13R (5′-CAGGAAACAGCTATGACC-3′) and M13F (5′-TGTAAAACGACGGCCAGT-3′). Thereafter, the nucleotide sequences of the *HSP70* gene obtained from sand fly isolates were compared to sequences obtained from dog and human *Leishmania* isolates from the same focus and to published sequences from other parasite species using Clustal X, version 1.83 [18]. Molecular Evolutionary Genetics Analysis software version 3.0 (MEGA3) was used for phylogenetic analysis [19].

### Host Blood Meal Analysis

During the 2010 field trip, 39 blood-fed wild-caught female sand flies were collected for host blood meal analysis by PCR. The head and tip of the abdomen of each fly were removed and used to identify the fly to species. The midguts, with blood meal intact, were removed and squashed onto a Whatman Clone Saver card (Whatman plc Springfield Mill, Kent ME14 2LE, UK) to preserve them for later analysis. Each preserved blood meal was labeled with the collection date, collection method (light trap or sticky trap), collection site and species identity. PCR-based identification of the mammalian blood was carried out according to the method of Kent and Norris [20]. DNA was isolated from punches of the Clone Saver card using the QIAmp DNA Micro kit (Qiagen Inc., Valencia, CA) according to the manufacturer's protocol and the DNA was eluted in 20 µL of ultrapure H_2_O. Due to the variability in the size of the bloodmeal of field-collected samples, the integrity of the DNA was verified using universal vertebrate-specific primers. Separate PCR reactions were prepared for the amplification of dog, human or universal vertebrate-specific cytochrome b. Each 25 µL reaction mixture consisted of 12.5 µL of PCR Master (Roche Applied Science, Indianapolis, IN), 200 pmol of Human741F, Dog 368F, or UNFOR403 and UNREV1025 primers, 2.5 µL RediLoad (Invitrogen) and approximately 15 ng DNA template. The reaction was initiated with a 5-minute denaturation at 95°C followed by 35 cycles at 95°C for 1 minute, 58°C for 1 minute and 72°C for 1 minute. The extension step was performed at 72°C for 7 minutes. Amplicons were produced in a GeneAmp PCR System 9700 (Applied Biosystems, Carlsbad, CA) thermocycler and the products visualized on cyanine-stained 1.5% agarose gel alongside a TrackIt 100 bp DNA ladder (Invitrogen, Carlsbad, CA). Positive control DNA templates were extracted from *Phlebotomus perniciosus* fed artificially through a membrane on dog blood and *Phlebotomus duboscqi* fed on a human and were processed in a manner identical to field-caught specimens.

## Results

### Vector Surveillance

#### Sand fly species diversity and relative abundance

**Figure 2:**
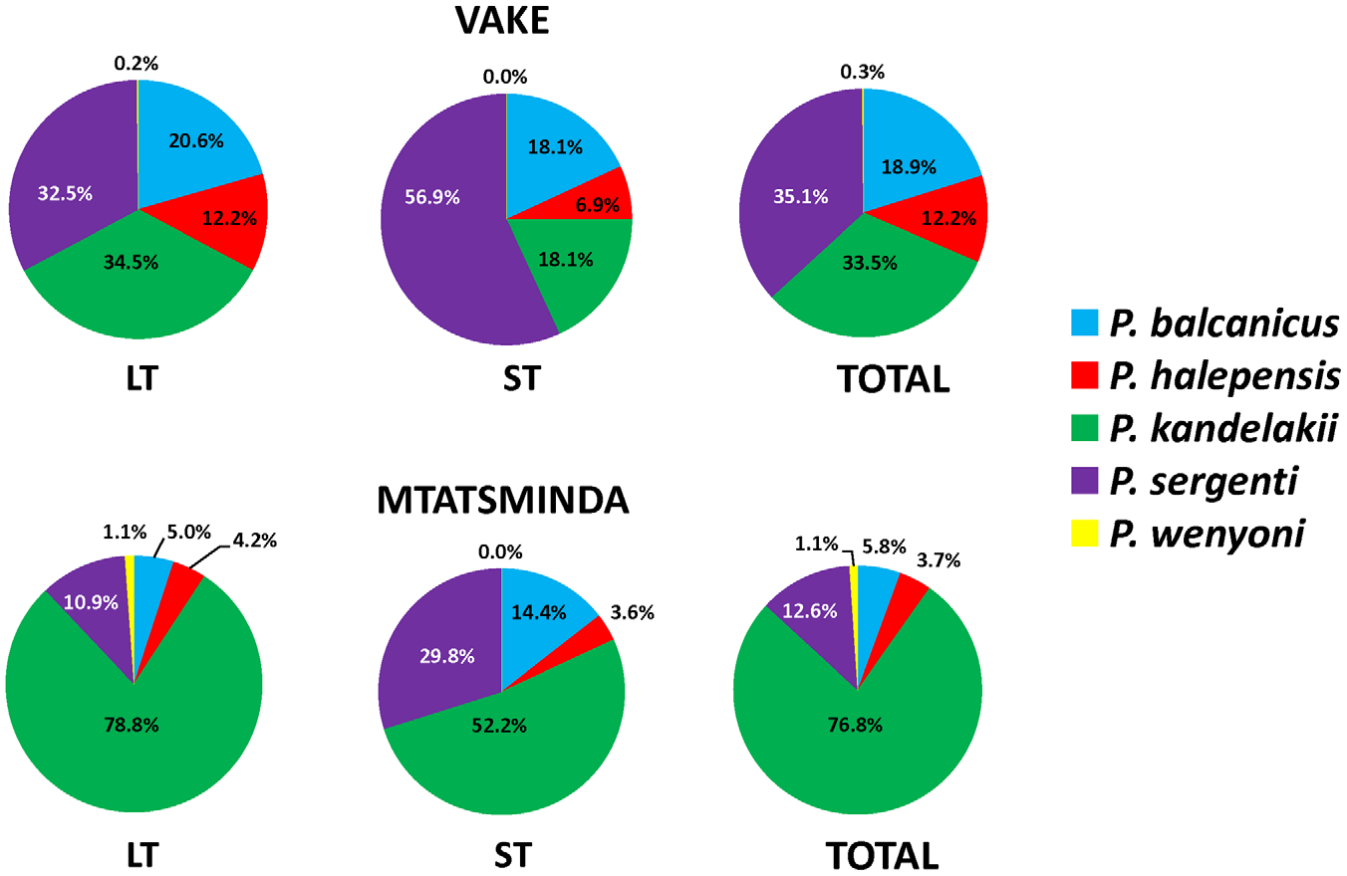
Diversity and relative abundance of sand fly species collected in Vake and Mtatsminda districts, 2006–2008. LT = light trap; ST = sticky trap.

A total of 1,266 male and 1,179 female sand flies were collected in routine surveys from the two suburban communities of Vake and Mtatsminda districts during the period 2006–2008, comprising five *Phlebotomus* species of three subgenera as follows: *P. balcanicus* and *P. halepensis* of the subgenus Adlerius; *P. kandelakii* and *P. wenyoni* of the subgenus *Larroussius*; *P. sergenti* of the subgenus *Paraphlebotomus* (Table 1). Whereas all five species were prevalent in both Vake and Mtatsminda, some significant differences in relative abundance were observed between the two communities. The predominant species in Vake was *P. sergenti*, followed in descending order by *P. kandelakii*, *P. balcanicus*, *P. halepensis*, and *P. wenyoni*. In Mtatsminda, *P. kandelakii* comprised over three fourths of the total sand flies collected, followed by *P. sergenti*, *P. balcanicus*, *P. halepensi*s, and *P. wenyoni* (Table 1, Figure 2). Apart from *P. wenyoni*, all species were well represented by light trap and sticky trap collections, the two trapping techniques used in this study. Overall, the number of sand flies captured by light traps in routine collections was 8.9-fold higher than captured by sticky traps. The fold increase was calculated by dividing the total number of flies captured in routine light trap collections (2035) by the total number of flies captured on sticky traps in parallel routine collections at the same sites on the same nights (228). By community, 5.1-fold and 12.3-fold more sand flies were captured by light traps than by sticky traps in Vake and Mtatsminda, respectively. The ratio of males to females in light trap collections was 0.795 and 0.919 for Vake and Mtatsminda, respectively. Corresponding ratios in sticky trap collections were 0.229 and 0.240. In Mtatsminda, the order of abundance by species was the same in light trap and sticky trap collections (Figure 2), but in Vake the relative proportion of *P. kandelakii* flies caught by light traps was about two fold greater than caught by sticky traps. Interestingly, the opposite was observed for *P. sergenti* where the relative proportion was1.8 fold greater on sticky traps (Figure 2).

**Table 1:** Diversity and relative abundance of sand fly species in Vake and Mtatsminda districts.

Sand fly species	Vake				Mtatsminda				Total			
	M	F	T	%	M	F	T	%	M	F	T	%
*P. balcanicus*	60	63	123	18.9	51	53	104	5.8	111	116	227	9.3
*P. halepensis*	69	10	79	12.2	51	15	66	3.7	120	25	145	5.9
*P. kandelakii*	101	117	218	33.5	679	700	1379	76.8	780	817	1597	65.3
*P. wenyoni*	2	0	2	0.3	13	6	19	1.1	15	6	21	0.9
*P. sergenti*	133	95	228	35.1	107	120	227	12.6	240	215	455	18.6
**Total**	365	285	650	100	901	894	1795	100.0	1266	1179	2445	100.0

Collections were made from May 2006 through September 2008. M = male; F = female; T = species total.

### Spatial and Temporal Distribution

**Figure 3:**
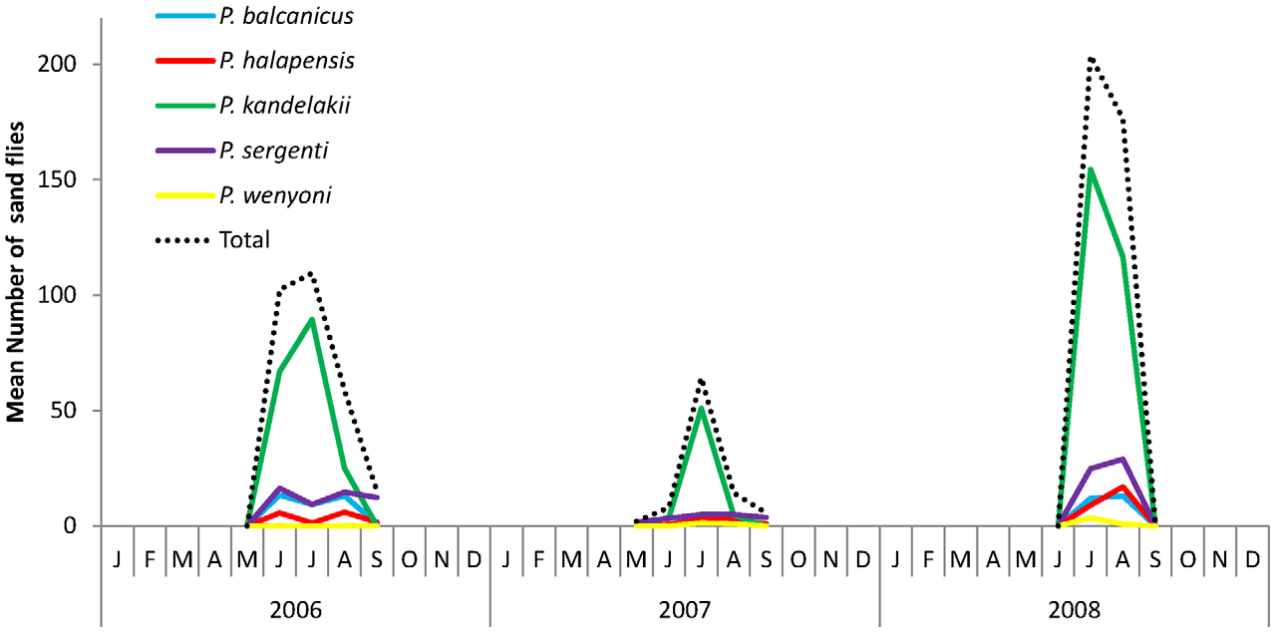
Seasonal distribution of sand fly species collected from Vake and Mtatsminda districts, 2006–2008.

Sand flies were collected in abundance in and around case sites, especially near chicken coops and animal pens, from early June through mid August during each of the survey years (Figure 3). Analysis of collection data over three summers clearly reveals a short sand fly season and a unimodal annual distribution pattern, with sand fly emergence beginning around the first week of June and population densities increasing to a peak in July and then declining through late August to zero in early September (Figure 3).

### *Leishmania* Surveillance

**Figure 4:**
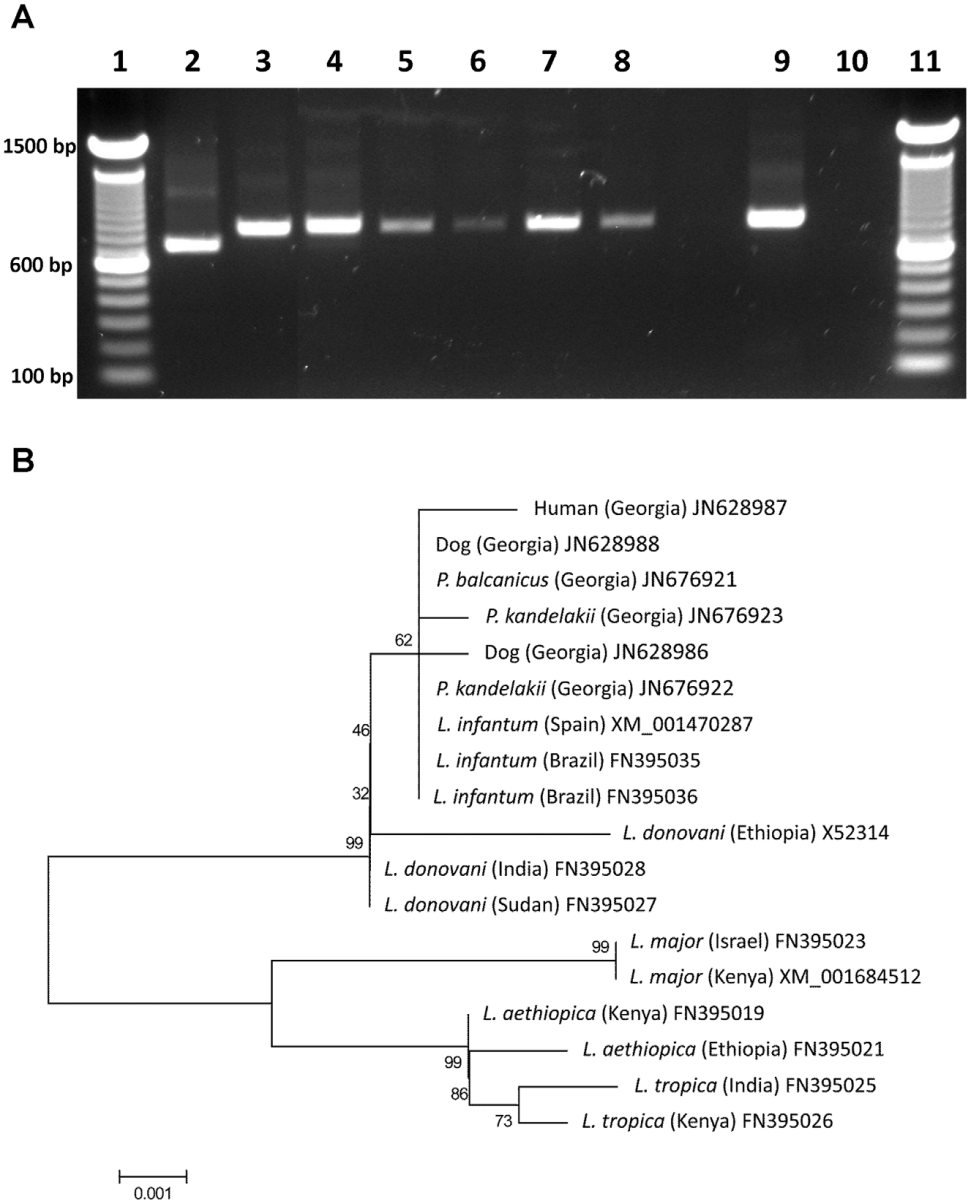
Molecular characterization of *Leishmania* isolates from wild-caught sand flies in the Tbilisi VL focus. (A) Lanes 1 and 11, DNA size marker (100 base pair ladder); lane 2, *L. major* (MHOM/IL/80/Friedlin); lane 3, *L. infantum* (MHOM/ES/00/UCM-1); lanes 4–8, *Leishmania* isolates from *P. kandelakii*; lane 9, *Leishmania* isolate from *P. balcanicus*; lane 10, negative control. bp = basepairs. (B) Phylogenetic analysis of the *Leishmania* 70 kDa heat-shock protein (HSP70) gene. The sequences are represented by the *Leishmania sp.*, country of origin in parentheses and GenBank nucleotide accession numbers. Node values indicate branch support.

In July–August of 2008, three of 283 (1.1%) female sand flies dissected and examined for *Leishmania*, were found infected. These included two of 223 *P. kandelakii* (0.9%) and one of 14 *P. balcanicus* (7.1%) (Table 2). All three flies harbored massive, mature infections with attachment to the microvillar lining of the midgut wall and to the cuticular intima of the stomodeal valve. Nectomonads and haptomonads were packed tightly in the thoracic midgut, behind the stomodeal valve, forming a “plug” with a high proportion of free-swimming metacyclic parasites that escaped into the dissection fluid and were distinguished by a small cell body, long flagella and rapid movement. These infectious forms were clearly visible under high magnification (40×). In some of the infections, the entire thoracic midgut was occluded with a plug of parasites that were mostly trapped in a gel matrix [21]. Similarly, in July of 2010, nine of 376 (2.4%) females dissected and examined were found infected, including eight of 320 *P. kandelakii* (2.5%) and one of 24 *P. balcanicus* (4.2%) (Table 2). PCR analysis of six successful isolates identified the parasites as *Leishmania* (Figure 4A). Further sequencing of the *Leishmania HSP70* from two *P. kandelakii* and one *P. balcanicus* isolates followed by sequence alignment against sequences from a human and two dog isolates from the same focus and other *Leishmania* species confirmed the parasite identity as *L. infantum* (Figure 4B). Of note, the sand fly isolates were identical to the human and dog isolates from the same focus (Figure 4B).

**Table 2:** Natural *Leishmania* infections in wild-caught sand flies from VL case sites in Tbilisi, Georgia.

Collection Year	2008		2010		Total	
Sand fly species	No. Dissected	No. positive (%)	No. Dissected	No. positive (%)	Total Dissected	No. positive (%)
*P. balcanicus*	14	1 (7.1%)	24	1 (4.2%)	38	2 (5.3%)
*P. halepensis*	11	0	11	0	22	0
*P. kandelakii*	223	2 (0.9%)	320	8 (2.5%)	543	10 (1.8%)
*P. wenyoni*	1	0	1	0	2	0
*P. sergenti*	35	0	20	0	55	0
**Total**	284	3 (1.1%)	376	9 (2.4%)	660	12 (1.8%)

### Host Blood Meal Analysis

**Figure 5:**
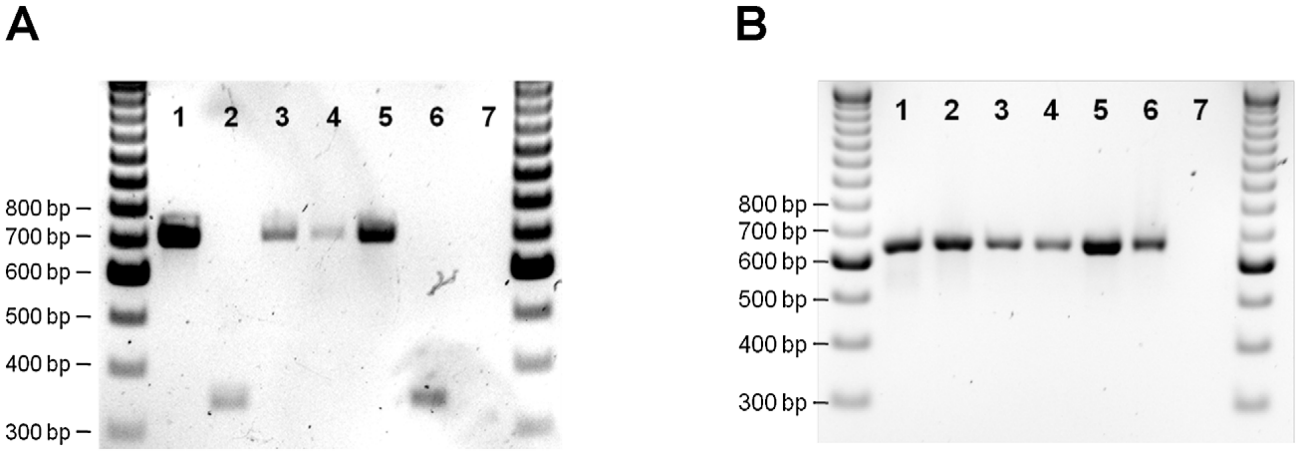
Analysis of blood meals from wild-caught sand flies in the Tbilisi VL focus. Cyanine-stained agarose gel showing (A) dog- or human-specific and (B) universal cytochrome b amplicons from polymerase chain reactions containing DNA extracted from blood-fed phlebotomine sand flies. Control products are shown in lane 1 (*Phlebotomus perniciousus* fed on a dog) and 2 (*Phlebotomus dubosqi* fed on a human). Products of representative samples of field-collected blood-fed *Phlebotomus kandelakii* are shown in lanes 3–6. Lane 7, negative control. The outside lanes are 100 basepair DNA ladders. bp = basepairs.

Of 39 blood-fed *Phlebotomus* sand flies collected from the Mtatsminda district of Tbilisi, Georgia, 27 were suitable for analysis by PCR amplification of the cytochrome b gene. As *L. infantum* infection is propagated between dogs, the main infection reservoirs, and humans by sand flies, it was important to identify which of the blood-fed flies fed on either dog (730 bp) or human (360 bp) blood (Table 3, Figure 5A). A universal cytochrome b target was used as a DNA quality control (Figure 5B). *Phlebotomus kandelakii* was the most abundant sand fly species collected, comprising 25 of the 27 blood-fed females available for analysis (Table 3). The remaining two blood-fed specimens were identified as *P. halepensis* and *P. sergenti*. There were no blood-fed *P. balcanicus* captured. The host source was identified in 80% of the 25 *P. kandelakii* blood meals. Dogs were identified as the preferred host (76%) of *P. kandelakii* and were the source of blood for each of the fed *P. halepensis* and *P. sergenti* specimens (Table 3). A human blood meal was identified in a single *P. kandelakii* sand fly (Figure 5A). Five *P. kandelakii* sand flies contained blood meals that were not identified as dog or human but were confirmed as vertebrate hosts by universal amplification of cytochrome b. Mixed dog/human blood meals were not detected.

**Table 3:** Blood meal sources of *Phlebotomus* sand flies from VL case sites in Tbilisi, Georgia.

Sand fly species	Host species			
	Total tested	Total identified	Human	Dog
***P. kandelakii***	25	20	1 (4.0%)	19 (76.0%)
***P. halepensis***	1	1	0	1
***P. sergenti***	1	1	0	1
***P. balcanicus***	0	0	0	0
**Total**	27	22	1 (3.7%)	21 (77.8%)

## Discussion

Fourteen *Phlebotomus* species have been reported from the eastern part of Georgia, most in mountainous rural or periurban areas [11]. The rather low species diversity observed in the current study may be a reflection of the peri-urban environment in which they were collected and emphasizes the urban nature of the Tbilisi focus where sand flies are likely breeding within or close to human habitation.

Of the species collected in this study, those of the subgenera *Larroussius* and *Adlerius* were of particular interest as potential vectors. Species of these subgenera have been incriminated or implicated elsewhere as vectors of *L. infantum* [5], [7]–[10], [22]. *P. (Larroussius) kandelakii* is a proven vector of *L. infantum* in Iran [22] and *P.* (*Adlerius*) *balcanicus* is a suspected vector of *L. infantum* in Greece and Serbia [23].

In the Old world there is a noticeable association between *Leishmania* species or complexes and particular subgenera of *Phlebotomus* [24]. Thus, it is reasonable to suspect putative vectors in previously unexplored foci based on their close taxonomic relationship to known vectors. Killick-Kendrick [5] noted that most incriminated vectors of *L. infantum* belong to the subgenus *Larroussius*. Phylogentically, the subgenus *Adlerius* is closely related to this subgenus [25] but most studies have not fully incriminated members of the subgenus *Adlerius* as vectors.

Routine vector surveillance in the suburban communities of Vake and Mtatsminda revealed an abundance of sand flies during a remarkably short season spanning early June through early September, suggesting a univoltine population that undergoes an obligatory or facultative diapause that carries them through the fall, winter and spring months. This is consistent with the findings of earlier workers who studied the biology of sand flies in foci of VL in mountainous areas of eastern Georgian USSR from 1945–1952 [1]. Diapause appears to be a common strategy in temperate sand fly species for surviving unfavorably cold conditions. For example, *Phlebotomus ariasi* in the Cevennes region of southern France undergoes a facultative diapause triggered by lower temperatures and a shorter photoperiod at the end of a short summer season [26], [27]. Other species undergo obligatory diapause triggered by diminishing photo period length [14]. In light of the shortness of the sand fly season, the high seroprevalence in children and dogs living in this focus [4] is indicative of a highly efficient transmission cycle. This is perhaps sustained by the presence, as established in this study, of more than one vector species transmitting and spreading the infection. The expansion and emergence of new foci of *L. infantum* in Georgia may also be accounted for by the breakdown of surveillance and vector control efforts in areas where the infection is prevalent.

The overall infection rate reported in this study (1.8%) is about half that reported by Lemer [1] over six decades ago. However, the promastigotes (referred to as leptomonads) in the earlier study were not identified as *Leishmania* nor was the feeding status of the infected flies determined. Killick-Kendrick and Ward [5], [28] outlined five criteria that should be fulfilled before a sand fly species is incriminated as a vector of human leishmaniasis with reasonable certainty: 1) Overlap of the geographic distribution of the suspected vector and human cases; 2) Sufficient abundance of the suspected vector necessary to maintain transmission; 3) Mature infections in naturally or experimentally infected flies; 4) Experimental transmission by bite; and 5) Isolation of *Leishmania* from wild-caught sand flies indistinguishable from the parasite causing disease in humans in the same place. These are highly stringent criteria and in the case of *P. kandelakii*, all have been satisfied apart from experimental transmission. This includes the first isolation and characterization of live *L. infantum* parasites from this species. With the advent of PCR technologies there has been a tendency by some to incriminate vectors based solely on the presence of *Leishmania* DNA. However, because a non-vector may imbibe blood from an infected host and thereby ingest *Leishmania* parasites, such findings, particularly in the absence of careful assessment of the feeding status, must be interpreted with caution. For *P. balcanicus*, the three most relevant incrimination criteria (the above-mentioned 1, 3, and 5) were also met, and while *P. kandelakii* was more prevalent in collections throughout the study period, the higher infection rates observed in *P. balcanicus* in 2008 and 2010 indicate that both are competent vectors of *L. infantum* in the Tbilisi focus. The finding in this study of massive, mature infections with a high proportion of metacyclic *Leishmania* parasites in both *P. kandelakii* and *P. balcanicus* is a demonstration of their natural ability to harbor the parasites through their complete extrinsic life cycle. More extensive trapping is needed to determine whether *P. halepensis* and *P. wenyoni* are also involved in parasite transmission in Tbilisi.

VL due to *L. infantum* is a zoonosis where the parasites are circulated between canines, the reservoirs of the infection. Humans become infected as accidental or incidental hosts with dogs being the most relevant source of infection. Blood meal source identification clearly implicates dogs as the favored host for *P. kandelakii* and the best reservoir of *L. infantum* infection [29]. Additionally, finding one *P. kandelakii* specimen with human blood is epidemiologically relevant providing evidence that this species feeds on both dogs and humans and thus can potentially spread infections from dogs to humans. Unfortunately, the absence of *P. balcanicus* blood-fed flies from our collection did not allow us to determine its feeding behavior and whether its role is primarily to propagate the infection among dogs or whether, similar to *P. kandelakii*, it feeds on humans and thus is likely to also transmit the parasite from dogs to humans. In addition to dogs and humans, chickens, cats, rodents, rabbits and bovines were present in the compounds where sand flies were collected. These animals, though epidemiologically irrelevant, represent potential sources of blood for the sand flies and likely account for the five unidentified *P. kandelakii* blood meals.

In conclusion, finding mature infections containing a high proportion of metacyclic promastigotes in both *P. kandelakii* and *P. balcanicus* provides strong evidence that they are capable of harboring *L. infantum* through its complete life cycle. These findings enable us to declare with confidence that *P. kandelakii* and *P. balcanicus* are vectors of *Leishmania infantum* in the VL focus in Tbilisi, Georgia. Based on its high relative abundance, *P. kandelakii* is probably the primary vector. However, the higher percentage of natural infections observed in *P. balcanicus* indicates that it is the more efficient vector and therefore plays a significant role in the epidemiology of visceral *Leishmania*sis in this focus.
